# “*We chose PrEP because I wanted to be sure that this child my wife was going to conceive was indeed mine.”* Factors influencing the choice of safer conception methods and experiences with its use: a qualitative study among HIV sero-discordant couples in Zimbabwe

**DOI:** 10.1186/s12889-024-19155-9

**Published:** 2024-07-19

**Authors:** Joelle M. Brown, Petina Musara, Serah Gitome, Miria Chitukuta, Bismark Mataveke, Thandiwe Chirenda, Nyaradzo Mgodi, Prisca Mutero, Allen Matubu, Gift Chareka, Charles Chasakara, Caroline Murombedzi, Tinei Makurumure, Carolyn Smith Hughes, Elizabeth Bukusi, Craig R. Cohen, Stephen Shiboski, Lynae Darbes, George W. Rutherford, Z. Michael Chirenje, Felix Mhlanga

**Affiliations:** 1 Department of Epidemiology & Biostatistics, University of California San Francisco, San Francisco, CA USA; 2 Department of Obstetrics, Gynecology & Reproductive Sciences, University of California San Francisco, San Francisco, CA USA; 3 Institute for Global Health Sciences, University of California San Francisco, San Francisco, CA USA; 4 University of Zimbabwe Clinical Trials Research Centre, Harare, Zimbabwe; 5 Centre for Microbiology Research, Kenya Medical Research Institute, Nairobi, Kenya; 6 University of Zimbabwe, Harare, Zimbabwe; 7 Mercy-Care Fertility Centre, Harare, Zimbabwe; 8 Department of Health Behavior and Biological Sciences, University of Michigan, Ann Arbor, MI USA

**Keywords:** HIV prevention, PrEP, Zimbabwe, LMIC, Safer conception, HIV-discordant couples, sub-Saharan Africa, ART, Semen washing, Vaginal insemination

## Abstract

**Background:**

Safer conception services are needed to minimize HIV transmission among HIV sero-discordant couples desiring pregnancy. Few studies have evaluated the choices couples make when they are offered multiple safer conception methods or real-world method acceptability. This paper addresses an important knowledge gap regarding factors that influence the choice of safer conception methods, couples' actual experiences using safer conception methods, and why some couples switch safer conception methods.

**Methods:**

Between February and June 2019, we conducted semi-structured in-depth interviews among 14 men and 17 women, representing 17 couples who exited the SAFER study—a pilot safer conception study for HIV sero-discordant couples in Zimbabwe that offered couples a choice of ART with monthly viral load monitoring (ART/VL), oral PrEP, vaginal insemination, and semen washing. All couples in SAFER had used at least two safer conception methods.

**Results:**

We found that safer conception method choice often centered around a desire for intimacy, condomless sex, and certainty in the conception process, particularly for men. Method-related attributes such as familiarity, perceived ease of use, side effects, and perceived level of effectiveness in preventing HIV and achieving pregnancy influenced method choice, switching, and satisfaction. Concerns were expressed about each safer conception method and couples were willing to try different methods until they found method(s) that worked for them. The majority of participants reported having positive experiences using safer conception, especially those using ART/VL + PrEP, citing that they were able to attempt pregnancy for the first time with peace of mind and experienced joy and satisfaction from being able to achieve pregnancy safely.

**Conclusions:**

The differences in method preferences and experiences voiced by participants in this study and in other studies from the region point to the importance of having a variety of safer conception options in the service delivery package and addressing concerns about paternity, intimacy, and method-related attributes to enable HIV sero-discordant couples to safely achieve their reproductive goals.

**Supplementary Information:**

The online version contains supplementary material available at 10.1186/s12889-024-19155-9.

## Introduction

Across sub-Saharan Africa (SSA), the vast majority of people living with HIV (PLWH) are of reproductive age [1–3]. Advancements in antiretroviral therapy (ART) have significantly improved the quality of life of PLWH, and both the desire for children and pregnancy rates have increased [4–6]. It is estimated that among PLWH who are in stable relationships, up to one-half of partnerships include a partner without HIV [7, 8]. If HIV viral suppression is not achieved or sustained, these HIV sero-discordant couples (one partner is HIV-positive while the other is not) are at risk for HIV transmission to the HIV-negative partner [9, 10], particularly when having vaginal intercourse without a condom to achieve pregnancy [11–13]. Sero-discordant couples need support to attain their fertility goals safely without putting their negative partner at risk of HIV [11, 13, 14].

Safer conception interventions are a critical component of the support needed by HIV sero-discordant couples for them to conceive while minimizing the risk for HIV [12]. These interventions reduce HIV risk when adhered to and include ART with viral suppression for the partner living with HIV, pre-exposure prophylaxis (PrEP) for the partner without HIV, vaginal insemination for couples with a woman living with HIV and semen washing for couples with a man living with HIV [15–19]. The World Health Organization (WHO) recommends ART for all people living with HIV and PrEP for sero-discordant couples. While ART has been scaled up in SSA and oral PrEP is increasingly available in some settings, they are generally not provided in the context of safer conception programs and the implementation of safer conception services has largely been limited to research projects [20]. Furthermore, there are no global safer conception guidelines, and few countries in HIV-endemic settings have country-level safer conception policies [20–23], leaving many providers unprepared to counsel the vast majority of women and men in need of safer conception services [24].

To date, most research on safer conception in SSA has shown that couples would accept various safer conception methods if offered, although the majority of these studies asked about hypothetical use [20]. Data on the actual use of safer conception methods such as ART/VL, PrEP, vaginal insemination, and semen washing among sero-discordant couples with fertility intentions in SSA are limited [25–30]. To fill this important knowledge gap, we carried out a qualitative study among men and women who received safer conception services as part of a pilot study called SAFER [25] in Zimbabwe, a country that has been hit particularly hard by the HIV pandemic, with 1.2 million reproductive-age adults living with HIV and an estimated 25,000 new HIV infections per year [1]. In this study, we aimed to better understand couples’ decision-making regarding safer conception, including factors that affect method preferences and selection, experiences of use, and method switching. Our findings will help inform the development and delivery of safer conception services among HIV sero-discordant couples in Zimbabwe and similar settings.

## Methods

### Study design, population and data collection

Between February and June 2019, we invited all 46 participants who had exited the SAFER pilot study (Table 1) to participate in semi-structured in-depth interviews (IDIs) to explore factors that influenced method choice, experiences using the methods, and reasons for switching methods. We developed a semi-structured IDI guide (available in Appendix 1), drawing on the Theoretical Framework for Acceptability [31], to frame the discussion of relevant themes and sufficient flexibility to allow for unexpected discoveries of social processes and cultural meanings [32]. The IDI guide included open-ended questions on safer conception decision-making, factors influencing the choice of safer conception methods, and experiences using safer conception strategies and services. IDI questions were developed to ensure that the study aims were achieved, and the questions were refined after pilot testing. Face-to-face, semi-structured IDIs were administered by three experienced, gender-matched social scientists trained on the protocol but who were not involved in the SAFER pilot study. For quality control, each interviewer conducted 1–2 supervised mock interviews and received feedback and approval from the study’s senior social scientist (PeM) prior to collecting the actual data. Members of couples were interviewed separately. All interviews were conducted in a private room located within the University of Zimbabwe-Clinical Trials Research Centre (UZ-CTRC) study site situated on the grounds of the Zengeza Municipality Clinic in Chitungwiza, Zimbabwe. IDIs were conducted in the participant’s preferred language, either Shona or English, and each lasted 1–2 hours, depending on the time participants needed to answer the questions. IDIs were audio-recorded with consent from the participants; the audio files were transcribed and, if needed, translated into English. Sociodemographic data on the SAFER participants were collected during the SAFER study. The detailed methods of the SAFER study and its clinical findings are presented in Brown et al*.* [25] and summarized in Table 1.

**Table 1:** Description of the SAFER pilot study in Zimbabwe

The SAFER study was a prospective, nonrandomized pilot study to measure the uptake, acceptability, cost-effectiveness, and impact of multiple safer conception strategies among 23 HIV sero-discordant couples in Chitungwiza and Harare, Zimbabwe, conducted between March 2017 and June 2019 [25]. To be eligible for SAFER, participants were part of a heterosexual HIV sero-discordant relationship, men were 18+ years of age, women were 18-35 years, they were sexually active, and they were seeking pregnancy in the next 6 months. The SAFER study was implemented by the UZ-CTRC on the grounds of the Zengeza Municipality Clinic in Chitungwiza, Zimbabwe.
All couples participating in the SAFER study:
• One or more currently available safer conception methods were chosen: antiretroviral therapy (ART) with monthly viral load (VL) monitoring for the HIV-positive partner (ART/VL), oral preexposure prophylaxis (PrEP) for the HIV-negative partner, vaginal insemination (VI) for couples with an HIV-positive woman, and semen washing (SW) for couples with an HIV-positive man.
• Received counseling on HIV prevention and the use of safer conception methods, guided by a safer conception counseling toolkit specifically developed for healthcare providers, offers safer conceptions to HIV sero-discordant couples in SSA [33]. (Available at: http://www.hiveonline.org/safer-conception-toolkit-for-hiv-affected-individuals-and-couples-and-healthcare-providers/)
• Prior to conception attempts, all enrolled couples underwent a two-month run-in period during which they returned to the clinic each month to receive additional information and counseling on safer conception methods, as needed. Upon completion of the run-in period, couples were counseled to begin conception attempts with their chosen safer conception method(s).
• Couples were followed monthly for up to 12 months of pregnancy attempts, quarterly during pregnancy, and 12 weeks postpartum. At each visit, data on method use, urine for pregnancy testing, blood for HIV antibody testing, or viral load, if HIV-positive, were obtained. Infants born to HIV-positive women were tested for HIV at 6 and 12 weeks.
At enrollment, all couples in SAFER chose ART/VL, and all couples chose at least one additional method; 74% chose PrEP, 36% chose SW, and 25% chose VI. During prepregnancy follow-up visits, three couples discontinued SW, and one couple discontinued VI; all four of these couples opted for ART/VL+PrEP. Twelve couples achieved pregnancy. There were no cases of HIV transmission to partners, and no infants tested positive for HIV.

### Data analysis

All IDI transcripts were independently reviewed and coded by two investigators (PeM, MC) using a codebook that was developed after the interviews, reviewed, and tested by study investigators and research team members using the first completed transcripts. During the analysis period, two coders (PeM and MC) met weekly to discuss the applied codes and emerging themes. To check for consistency in text interpretation during this process, coding was compared across the coders using an agreed-upon codebook, and discrepancies were discussed by the research team (PeM, MC, JMB, SG and FM) until resolution. Overall, the level of intercoder reliability was approximately 80%. Code reports were run for the following codes: choice of methods, use experience, barriers and facilitators to use, method preferences and future pregnancies. The code reports were summarized into memos, the dominant themes were organized, and representative quotes were chosen to illustrate these themes in the words of the participants. DEDOOSE Software Version 9 (SocioCultural Research Consultants, LLC, Los Angeles, California) was used for data management, organization, and coding. The COREQ checklist was used for reporting the study findings [34].

### Ethical considerations

The study protocol, consent forms, interview guides, and all participant-related materials were approved by the University of California, San Francisco Institutional Review Board, the Municipality of Chitungwiza, the Medical Research Council of Zimbabwe, the Medicines Control Authority of Zimbabwe, and the Research Council of Zimbabwe. Each couple member provided consent individually in their preferred language (English or Shona) to minimize coercion and provided written informed consent prior to study participation. All IDI study participants were reimbursed USD10 for their time and transport expenses.

## Results

We conducted IDIs among 31 participants (17 women (7 living with HIV) and 14 men (8 living with HIV)) who exited the SAFER study. These 31 participants represented 17 couples; 14 couples with both dyad members interviewed and three couples with only one member interviewed. Fifteen participants declined to participate in IDIs because they were unavailable due to work commitments (*n* = 3) or uninterested in further participation due to relationship dissolution (*n* = 4) or other unspecified reasons (*n* = 8). The median age of the women was 32 years (range: 21–35), and that of the men was 34 years (range: 24–54) (Table 2). Approximately three-quarters of men and two-thirds of women had completed secondary education. Twelve of the 17 women (71%) achieved pregnancy. All couples had chosen to use a combination of at least two safer conception methods during SAFER; all (100%) couples chose ART/VL, and all couples chose at least one additional method.

**Table 2:** Characteristics of male (*n*=14) and female (*n*=17) participants interviewed

	**Men**	**Women**
	**Median (IQR) or n (%)**	
Age in years (range)	34 (32–49)	32 (28–33)
Completed secondary education (%)	11 (78.6)	11 (64.7)
Married to and living with study partner (%)	14 (100)	17 (100)
Employed (%)	13 (92.9)	8 (47.1)
HIV-positive (%)	8 (57.1)	7 (41.2)
Months on ART if HIV-positive (range)	36 (8–70)	15 (3–33)
Parity (range)	–	2 (0–3)
1 or more living children with current partner (%)	9 (64.3)	10 (59.9)
Number of living children, total (range)	2 (0–4)	2 (0–4)

*IQR *interquartile range, *ART *antiretroviral therapy

**Fig. 1:**
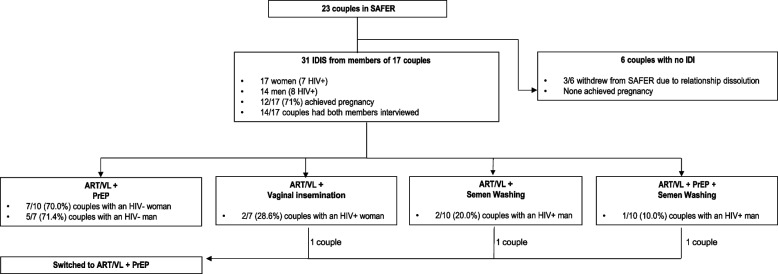
Safer conception method uptake and switching among those interviewed

Among those interviewed (Fig. 1), PrEP was chosen by 12/17 couples (76.5%), including 7 HIV-negative women and 5 HIV-negative men. Two (28.6%) of 7 couples (28.6%) with HIV-positive women chose vaginal insemination. Three (30.0%) out of 10 couples with an HIV-positive man chose semen washing. Three couples switched safer conception methods during follow-up. One couple discontinued vaginal insemination, and two couples discontinued semen washing; all three couples switched to ART/VL + PrEP. The 6 couples that did not participate in IDIs were similar to those who did participate in terms of sociodemographics, method use, and method switching but three of them withdrew from SAFER due to relationship dissolution before pregnancy attempts, and none achieved pregnancy. Notably, no HIV-negative partners or infants tested positive for HIV during SAFER.

Three overarching themes related to the choice of safer conception methods emerged. First, safer conception method choice often centered around paternity concerns and the desire to have control over the conception process, including condomless sex, especially among men. Second, method-related attributes such as familiarity, perceived ease of use, side effects, and perceived level of effectiveness in preventing HIV and achieving pregnancy influenced method choice and switching. Third, couples' actual experiences with safer conception were generally positive, especially for those using ART/VL + PrEP, and were influenced by how easy it was to use and whether they achieved pregnancy. Below, we discuss these main themes and their subthemes in greater detail and provide illustrative quotes (see Appendix 2 for additional supporting quotes).

### Control over conception, paternity and condomless sex

One of the most common reasons given for selecting a safer conception method was the need to have control over the conception process, which was expressed as being able to ensure paternity and condomless sex during fertile days.

*“One of the things that made me to use this method …is that we will be in control of the process to have a child using that method.”*

(Man, HIV-negative, couple used ART/VL+PrEP)

Some participants were greatly concerned about the possibility that sperm could get switched in the lab during semen washing, which would raise doubts about whether the male partner was the biological father of the child. These perceptions were heavily influenced by gender, as male partners expressed a much stronger desire to have control over the conception process and ensure paternity compared to the female partners. Concerns about paternity were mostly raised by couples who selected ART/VL + PrEP, and given as the reason for not selecting semen washing.

*“Unprotected sex with my partner would make me feel better unlike collecting semen from me and washing it then injecting my wife ….. we chose PrEP because I wanted to be sure that this child my wife was going to conceive was indeed mine and for that to happen there … should be unprotected sexual intercourse between my wife and l, that is what would make us accept that the products of my wife’s womb will be surely mine unlike in a situation where semen washing is used, the probability of there being someone else’s semen planted will be there and l will never fully believe that the child will be mine….”*

(Man, living with HIV, couple used ART/VL+PrEP)

### Method-related attributes informing method selection

#### Familiarity and reassurance

Most participants with HIV were already taking ART prior to joining the SAFER study, and all HIV-positive participants opted to use ART with monthly viral load testing as a safer conception method. HIV-positive participants reported that the major advantage of monthly viral load testing was receiving frequent, ongoing reassurance that they were virally suppressed and, therefore, would remain healthy and protect their partner and newborn from HIV. Participants viewed PrEP as a “familiar” method in that it is a pill, which most of them said they were used to. In contrast, some steered away from vaginal insemination and semen washing because they felt that they were “unfamiliar methods” compared to taking pills (e.g., ART and PrEP).

*“As for the syringe one [vaginal insemination] we were not used to it, so we decided that let us do what we are used to, which are the pills, since there are pills that can prevent [HIV]… we have never used the syringes before.”*

(Woman, living with HIV, couple used ART/VL+PrEP)

#### Ease of use

Many couples opted for a safer conception method that they perceived as ‘easy to use’. Ease of use was defined as being ‘simple’ and ‘easy to understand’ and as not involving complicated procedures or special facilities, such as laboratories. For these reasons, many couples chose ART/VL + PrEP over semen washing and vaginal insemination.

*“We felt that it [ART/VL*+*PrEP] was an easy method that we agreed upon and we felt we were in control because some of the methods were a bit complicated for example the semen washing, after extracting the semen it needed to be quickly transported to the laboratory and other processes done, I felt that my wife was not going to be comfortable with the process.”*

(Man, living with HIV, couple used ART/VL+PrEP)

Most of the couples who did not select vaginal insemination perceived the procedures to be ‘difficult’ or ‘complex’. Men and women lacked confidence in their ability to use syringes to draw ejaculate from the condom and insert it into the woman.

*“The other methods were difficult for us, like the one that you would put your sperms in a syringe [vaginal insemination] then insert to your wife, for me the process of ejaculating your sperms and then inserting into the woman was a difficult process…”.*

*(*Man, HIV-negative, couple used ART/VL+PrEP)

#### Perceived effect on chances of becoming pregnant

The perceived effect of a safer conception method on the couple’s chances of becoming pregnant was observed to be an important consideration for couples. Some participants expressed that safer conception methods, such as semen washing, might speed up conception, while vaginal insemination might ‘delay the conception process’.

*“My mind just thought that during the time the sperms travel naturally, it’s slow, this one [semen washing] is fast in that they just inject them directly and you immediately become pregnant”.*

(Woman, HIV-negative, couple used ART/VL+SW, switched to ART/VL+PrEP]

#### Perceived level of effectiveness against HIV

Couples expressing the greatest concern about HIV transmission risk during condomless sex selected semen washing and vaginal insemination and doubted that PrEP would offer complete protection. HIV-positive men who chose semen washing strongly felt that they had the responsibility to protect their HIV-negative partner from HIV.

*“It is unfortunate that I got [HIV-]infected but I had told myself that I do not want to put her in a situation where she becomes vulnerable to contract HIV. That is why I chose semen washing because l know they will wash the semen before injecting it into her body.”*

(Man, living with HIV, couple used ART/VL+SW)

*“I didn’t believe that these tablets [PrEP] could help if you are having unprotected sex. You are at risk [of HIV]”*

(Man, HIV-negative, couple used ART/VL+VI, switched to ART/VL+PrEP)

Despite some initial scepticism about the effectiveness of PrEP, several participants who had chosen semen washing and vaginal insemination later switched to PrEP because they were unsatisfied with these methods. Those who initially selected PrEP, however, believed that they would be protected from HIV.

*“At the beginning we chose it [vaginal insemination] we were saying I think, it is safe, because there is no any semen that will get in contact with me or even him.*

(Woman, living with HIV, couple used ART/VL+VI, switched to ART/VL+PrEP)

#### Moral support

For some couples, safer conception was seen as a way of expressing moral support to the partner who was HIV-positive. This was particularly true for those participants who chose PrEP; the act of both partners taking pills was seen as a show of solidarity, especially for the HIV-positive partner who was already shouldering the burden of a long-term illness and chronic medication, and as an opportunity to support each other with adherence. In addition, the partners could remind each other to take their pills as prescribed, thus enhancing adherence.

*“I chose PrEP because it was good for me in that I was taking my PrEP, which are pills right. So, I would say when my husband would be taking his medication, which are pills as well. It just became similar. We are taking them at the same time.*”

(Woman, HIV-negative, couple used ART/VL+PrEP)

#### Side effects and stigma

A few participants expressed concerns about the possible side effects of PrEP, which led them to choose either vaginal insemination or semen washing. Some participants expressed concern about possible stigma related to taking antiretrovirals, and others did not agree that HIV-negative people should take antiretrovirals.

*“So I said ‘alright then take … the pills [referring to PrEP] and he said, ‘no I can’t, to take the pills when I don’t have the disease [HIV].”*

(Woman, living with HIV, couple used ART/VL+VI, switched to ART/VL+PrEP)

### Participant experiences using safer conception

#### Joy, lack of fear

Overall, the majority of participants reported having good experiences using safer conception methods, especially those using ART/VL and PrEP. They viewed safer conception methods as allowing them to attempt to become pregnant for the first time, for as long as possible, without fear. Participants derived joy and satisfaction from being able to conceive safely.

*“For me, the most important thing is the child that I have. That is what I can mention first. What was good was that, I got a chance to conceive without being afraid that ‘Eish now that I have had sex with my partner, maybe I have infected him’. It is difficult when you know that you are positive and your partner is not.”*

*(*Woman, living with HIV, couple used ART/VL+PrEP)

*“… we did semen washing and were successful. I was able to have a child and my wife remained negative. My wife now has joy that had been dampened by my testing HIV-positive but ever since we came here, she is now more comfortable.”*

(Man, living with HIV, couple used ART/VL+SW)

#### Frustration when pregnancy is not achieved

Across all method choices, couples found it challenging when they did not become pregnant, and this was a source of frustration as they tried to determine what had caused them to fail to become pregnant.

*“But the problem that we encountered was that to get pregnant. That is what was difficult.”*

*(Woman,* HIV-negative, couple using ART/VL+PrEP)

#### Experiences and challenges with ART and PrEP

Most couples using ART/VL and PrEP found it ‘easy to use’ or ‘convenient’, especially when traveling. Most couples found ART/VL to be familiar and acceptable, and valued receiving ongoing reassurance that they were being virally suppressed, with few reporting challenges.

*“Since I started the program, till we finished the program, they were always saying my viral load was suppressed and sometimes undetected”*

(Man, living with HIV, couple used ART/VL+PrEP)

Some couples reported that taking PrEP did not disrupt normal life, which was desirable. However, a few participants reported concerns about their own lack of adherence to ART and PrEP or their partners’ and worried that poor adherence would impact the method’s effectiveness and the development of drug resistance. Those taking PrEP attributed a lack of adherence to forgetfulness and not being used to regularly taking medication.

*“It [PrEP] was hard… because I never took any medication before. Yes, there were some days I missed. If I am not mistaken it should be about 4 or 5 [pills] for the whole process [study].”*

*(*Man, HIV-negative, couple used ART/VL+ VI, switched to ART/VL+PrEP)

Although the study staff discontinued one woman from PrEP due to serious side effects, most participants reported no side effects from using PrEP. This lack of side effects was cited by many participants as a positive experience while on PrEP.

*“I used PrEP, it’s a medicine that works well, it’s a method that you can actually trust, because I never encountered any risks, no pain, and those that they call, yes side effects of the medication, I never encountered them.”*

(Man, HIV-negative, couple used ART/VL+PrEP)

#### Experiences and challenges with tracking fertile days

Some men and women initially reported challenges with tracking fertile days, though these challenges were overcome with more experience, and couples supported one another in understanding and implementing this method.

*“ During the first days I was not good. I didn’t know it [referring to tracking fertile days] and my husband was the one who did it for me. So, I later understood it.”*

I: He taught you?

*R: Yes”*.

(Woman, HIV-negative, couple used ART/VL+SW, later switched to ART/VL+PrEP)

#### Experiences and challenges with using semen washing and reasons for switching

The major challenges reported with semen washing are related to the procedures involved. Some male participants were not comfortable with having to self-stimulate to produce the semen, citing that it is ‘difficult’. The fact that couples were aware that study staff were waiting to take the semen to the laboratory for washing seemed to worsen the discomfort and made it more difficult for the male partner to produce semen. This resulted in poor method satisfaction and, consequently, discontinuation and switching to ART/VL + PrEP, which they perceived to be easier to use.

*“It was just that issue of extracting, self-extraction of semen. The masturbation, yes. Haa, you would need, yah, eish, it would need magazines, whatever, a room whereby you will be following magazines. That masturbation process, it is a bit tricky. And people will be waiting for you. And everyone is aware and is waiting. So, you become a bit tense and what not”.*

(Man, living with HIV, couple used ART/VL+SW, switched to ART/VL+PrEP)

Some women reported discomfort with intrauterine insemination following semen washing, and procedures were reportedly time consuming and required specialized facilities, which also added to the challenges.

*“Then in semen washing, yes the strength is that it is very effective [for safer conception]. But it's time consuming… For the semen washing was a bit uncomfortable for me*. *Yes because the whole issue of opening the cervix. It was a bit uncomfortable but it was for the best.”*

(Woman, HIV-negative, couple used ART/VL+SW, switched to ART/VL+PrEP)

#### Experiences and challenges with using vaginal insemination and reasons for switching

Despite receiving training on how to carry out vaginal insemination, some couples reported dissatisfaction using this method, especially regarding the ‘complexity’ and the time involved. One participant described the experience with the method as ‘stressful’, as some of the semen spilled out of the vagina, and they worried that they would ‘fail’ to achieve pregnancy. One woman said the requirement to lie on the back after insemination was ‘tedious’ and ‘time consuming’, and having the male partner release the semen in a container was not ‘natural’. As a result, some couples stopped using vaginal insemination and switched to ART/VL + PrEP, which they perceived as easier to use.

*“Mmmm I can say we only faced a bit of challenge during the first month when we failed [to conceive]; we succeeded when we tried for the second time…you can take 30 minutes while so quiet, stuck in the house…people would come and knock, but it would appear as if no one is there it will be so quiet, 30 minutes sleeping with your back, until it’s done and then you get up and start doing your things.”*

(Woman, living with HIV, couple used ART/VL+VI)

#### Willingness to use safer conception methods for future pregnancies

Most couples who used ART/VL + PrEP expressed a willingness to use these same safer conception strategies for their future pregnancies, citing the positive experience they had with the methods. Couples were more likely to say that they would adopt the same method(s) if they found it easy to use and if they conceived while using it.

*“I will try this one, PrEP. I saw that it works. I tried it once and the following month she realized that she was pregnant.”*

(Man, HIV-negative, couple used ART/VL+VI, later switched to ART/VL+PrEP)

*“We did semen washing and we were successful. Semen washing is the one I will use [for future pregnancies].”*

(Man, living with HIV, couple used ART/VL+SW)

## Discussion

This study provides valuable insights into why HIV sero-discordant couples choose specific safer conception methods and their actual experiences using these methods in a real-world setting in Zimbabwe. Overall, we found that safer conception method choice often centered around a desire for control and certainty in the conception process, particularly for men. We found that method-related attributes, such as familiarity, ease of use, and perceived level of effectiveness in preventing HIV and achieving pregnancy, influenced method choice, switching, and satisfaction. Furthermore, we found that the majority of participants reported having positive experiences using safer conception, especially those using ART/VL + PrEP, citing that they were able to attempt pregnancy for the first time with peace of mind and experienced joy and satisfaction from being able to achieve pregnancy safely.

In our setting, most couples chose to use a combination of ART/VL + PrEP for safer conception. Being perceived as ‘familiar’ and ‘easy to use’ was important for those selecting these methods, which is supported by a similar study in Kenya [35]. In our setting, gender also played a key role in influencing the choice of these methods, with men expressing a strong desire to control the conception process and to ensure that they are the biological father. Men in particular viewed ART/VL and PrEP as allowing more ‘natural’, intimate, and condomless sex than semen washing and vaginal insemination. Our findings regarding men’s preferences for condomless sex and desire for control over the use of HIV prevention in general are well documented and consistent with other studies [36–39]. However, this underlying concern about paternity, which is specific to perceptions of semen washing, and the need for control in the context of safer conception among sero-discordant couples has not previously been documented and may warrant further investigation.

Semen washing and vaginal insemination were less frequently selected methods in our setting, and most couples who selected these methods later discontinued their use. While these methods are viewed as highly effective at preventing HIV, they alter the sexual experience because of the need for specialist facilities and equipment to conceive and raise concerns about paternity and the chances of conception. A recent study conducted in South Africa also revealed that semen washing was not a preferred method, as men felt it was an overly medicalized procedure to conceive and were concerned about paternity [40]. These findings suggest that safer conception programs should reflect the concerns and preferences of men and women in their target populations, and programs that align with gender and cultural norms will likely be met with greater acceptance.

In contrast to our findings, a similar study conducted in South Africa reported that PrEP was the least preferred method due to concerns about side effects and that vaginal insemination was the most popular option among HIV-positive women with HIV-negative partners [29]. This difference in method preferences suggests that there is no single safer conception method that will meet the needs of all sero-discordant couples. Safer conception programs may need to offer couples a range of method options to meet both couples' and individual partners' preferences and perceptions and should recognize that these preferences can change after using the methods and over time. We found that couples are willing to try different methods until they find one that works for them. Allowing couples to select their preferred safer conception methods may lead to increased uptake as well as adherence, as has been found in the contraceptive literature [41, 42].

The couples in this study had concerns about each safer conception method. Fears of side effects, doubts about effectiveness, and adherence challenges were most commonly reported for PrEP and ART, while complex procedures and a lack of control over conception and intimacy were major concerns for vaginal insemination and semen washing. These concerns have been reported in other similar studies [28, 35, 43]. Despite having received comprehensive counseling from study staff about each safer conception method on offer, some couples still, for example, did not trust that PrEP would protect them against HIV. Some couples did not select semen washing because of the fear that sperm could be switched by study staff during the process. In addition, some couples found the vaginal insemination procedure difficult. These concerns suggest areas where more intensive or frequent counseling, hands-on training sessions and supportive tools may be needed to enhance couples’ understanding of and skills to implement safer conception methods [44, 45], as well as the need to continue efforts to build trust between patients and healthcare providers. Doing so will enable couples and programs to benefit from a variety of safer conception methods. As noted above, these findings also reinforce the importance of offering options to couples and allowing them to choose the option(s) that best meet their needs.

This study has limitations. First, the generalizability of the study results may be limited, as the study was conducted at one research site in an urban research setting, and participant experiences may not be representative of those of the larger population of sero-discordant couples attempting pregnancy. Furthermore, all safer conception methods were provided for free and the findings may not reflect how out-of-pocket costs may influence uptake and use. A safer conception study in Kenya revealed that out-of-pocket costs were an important factor influencing the use and choice of safer conception methods among serodifferent couples [35]. Participants in the Kenyan study said they would prefer safer conception services and methods to be available to them for free in public clinics. Safer conception programs in resource-limited settings need to consider method acceptability as well as out-of-pocket costs for users, healthcare system costs and logistics, and the overall cost-effectiveness of safer conception programs in order to optimize country-level guidelines and service delivery.

Despite these limitations, the results of this study are supported by those of previous studies and have practical implications for the delivery of safer conception services in Zimbabwe and similar settings, especially given the dearth of information regarding perceptions of and experiences with safer conceptions in these areas. Furthermore, some of our unique findings have not been previously documented and diverge from those of other studies, thus providing rich insights that can inform the tailoring and inclusion of safer conception services in HIV treatment and prevention programming for couples in need.

## Conclusions

In conclusion, we found that concerns about paternity and method-related attributes, such as familiarity, ease of use, side effects, and level of effectiveness in preventing HIV and achieving pregnancy, influenced safer conception method choice, switching, and satisfaction. Methods that were easy to use and that allowed condomless sex and control over conception were more likely to be selected and used throughout the follow-up period. We found that the majority of participants reported having good experiences using safer conception, especially those using ART/VL + PrEP, and derived joy, peace and satisfaction from achieving pregnancy safely. The differences in method preferences and experiences voiced by participants in this study and in other studies from the region point to the importance of having a variety of options in the service delivery package for couples to be able to achieve their reproductive goals without transmitting HIV to the uninfected partner and baby.

### Supplementary Information

Supplementary Material 1. Appendix 1.

Supplementary Material 2. Appendix 2.

## Data Availability

The data that support the findings of this study are available from the corresponding author upon reasonable request.
