# Supplementary material for: “We chose PrEP because I wanted to be sure that this child my wife was going to conceive was indeed mine.” Factors influencing the choice of safer conception methods and experiences with its use: a qualitative study among HIV sero-discordant couples in Zimbabwe
Source: BMC Public Health. 2024 Jul 19;24:1936. doi: 10.1186/s12889-024-19155-9 (PMC11264618; doi:10.1186/s12889-024-19155-9)
Supplement: Supplementary file 1 — Supplementary Material 1. Appendix 1. [file 12889_2024_19155_MOESM1_ESM.doc]

Appendix 1. In-depth interview guide - topics relevant for this manuscript

In the next section I will ask you some questions about your experiences using safer conception. There are no right or wrong answers. We are trying to find out about your own experience so that programs can provide the best care.

1. **Please tell me about your experiences with safer conception services, with or without your partner.**

a. What safer conception strategies have you used (e.g. ART, VI, Semen washing, PrEP, fertility tracking)? How was that experience for you? What did you like about the safer conception strategy(ies) that you used? What did you not like about them? For the ones you did not choose, why did you not choose them?

b. If you and your partner chose more than one safer conception strategy, did you have a preference for one versus the other(s)? Why or why not?

c. Which strategies would you use for future pregnancies? Why would you use that one(s)? Which strategies would you never use? Why not?

d. How did you feel about keeping track of the fertile period? What did you like about it? What did you not like about it? Would you say tracking the fertile period fell more on you or your partner?

1. **Tell me about how you and your partner chose the safer conception strategies that you have used?**

a. What factors influenced your decision to choose the strategies that you ended up using?

1. **Can you tell me if you had any problems using your safer conception strategy? Or any problems continuing to use your safer conception strategy**?

Probes: What made it difficult to use your safer conception strategy? What made it easy for you to use your safer conception strategy? What things did you do to help you and your partner use your safer conception strategy? Can you tell me how safer conception services might be improved to make them easier to use and remember to use? Would you say that using the safer conception strategy fell more on you or your partner? Why was that?

1. **How do you feel about the amount of time it took to “use” safer conception services at your home?**

Probes: How did the use of safer services fit into your regular daily life? What aspects of using safer conception took too much time?

1. **How do you feel about the amount of time it took to travel to your clinic visits and receive your safer services?**

Probes: did you have to forgo any of your usual responsibilities to receive the safer services? (ie, work, household, or family duties) If yes, what was the impact for you or your family? What impact did it have on your ability to use the safer conception services or see your healthcare providers?

Thank you very much for your time.

That is the end of the interview, I really appreciate your willingness to speak to me about these issues.
