# Supplementary material for: “We chose PrEP because I wanted to be sure that this child my wife was going to conceive was indeed mine.” Factors influencing the choice of safer conception methods and experiences with its use: a qualitative study among HIV sero-discordant couples in Zimbabwe
Source: BMC Public Health. 2024 Jul 19;24:1936. doi: 10.1186/s12889-024-19155-9 (PMC11264618; doi:10.1186/s12889-024-19155-9)
Supplement: Supplementary file 2 — Supplementary Material 2. Appendix 2. [file 12889_2024_19155_MOESM2_ESM.docx]

**Appendix 2: Additional illustrative quotes**

| **Theme** | **Quote** |
| --- | --- |
| Control over conception process; ensure paternity | *Then there is this one, sperm washing, I asked my partner if we could use it and he said, “Ah what if I am given a child that is not mine”. Then we realized that we only had to use this one [PrEP]. It is the one he liked, the method of using PrEP and ART. Ah he wanted to ensure that he is the one who does it [gets me pregnant through sex] so that is why he liked this method.* (006F, HIV-positive woman, couple used ART/VL+PrEP) |
| Ease of use | *… we chose it [PrEP] because we saw that it is the one which is easy, which will not be hard on us…* (028F, HIV-negative woman, couple used ART/VL+PrEP) |
|  | *…that is why we chose a better option that both of us could easily understand until the end of the study.* (028M, HIV-positive man, couple used, ART/VL+PrEP) |
|  | *Yes. Aah, we just thought that they [referring to vaginal insemination] might be difficult for us. We might not be able to do what, [vaginal insemination]. Let’s just do what is easy for us.* (026F, HIV-positive woman, couple used ART/VL+PrEP) |
|  | *I didn’t understand that one [vaginal insemination] ….and my husband was not very keen on it. … I think that he didn’t understand how the insemination would work. He didn’t know if the process would be done when he is there, or he will do it himself and how he will collect [the sperm]*  (006F, HIV-positive woman, couple used ART/VL+PrEP) |
| Moral support | *So, if we say we wanted to use this [PrEP] method she would feel that she has someone who supports her.* (002M, HIV-negative man, couple used ART/VL+PrEP) |
|  | *I just chose [PrEP] because I didn’t want my wife to think that I was despising her and I said to myself let me use [PrEP].* (027M, HIV-negative man, couple used ART/VL+PrEP) |
| Perceived level of effectiveness | *I decided on it [vaginal insemination] because…the chance of contracting HIV would be limited.* (024M, HIV-negative man, couple used ART/VL+VI, switched to ART/VL+PrEP) |
|  | *When we discussed I said that maybe the sperm wash may not be effective for some reason and I end up getting infected with HIV. I found it easier to take [PrEP] while he takes ART and we track fertility. (025F, HIV-negative woman, couple used ART/VL+PrEP)* |
|  | *…Then my husband thought ‘Ah what if I take the [PrEP] pills and have unprotected sex with you, what if I contract the disease’.* (024F, HIV-positive woman, couple used ART/VL+VI, switched to ART/VL+PrEP) |
|  | *“I chose that one [PrEP] because it is the one I saw will be protecting my body”.*  *(*020F, HIV-negative woman, couple used ART/VL+PrEP] |
| Effect on chances of getting pregnant | *What I thought was the process [vaginal insemination] was going to delay the conception process, there might be low chances of getting pregnant”.* (011M, HIV-negative man, couple used ART/VL+PrEP) |
| Experiences and challenges using ART & PrEP | *I was already on ART. So, I never had any challenges”.* (018M HIV-positive man, couple used ART/VL+PrEP) |
|  | *I think it is only once, maybe in a year where you get [tested], what is that called, the viral load yes….But here [In safer study], it was a regular thing.* *(*007M, HIV-positive man, couple used ART/VL+SW, switched to ART/VL+PrEP) |
|  | *I liked that my life will remain where it is [remain HIV-negative]. My partner’s health will remain where it is, virally suppressed. (*020F, HIV-negative woman, couple used ART/VL+PrEP] |
|  | *But me I just saw that it [PrEP] is a strategy which just helped me. Even my mind became settled. Huh it then really became settled.* (026M, HIV-negative man, couple used ART/VL+PrEP) |
|  | *“I don’t have [challenges] even if I travel. If I want to go anywhere I know that my tablets I will have them so I don’t have any problem”.*  (026M, HIV-negative man, couple used ART/VL+PrEP) |
| Willingness to use safer conception methods for future pregnancies | *“We did semen washing and we were successful. Semen washing is the one I will use [for future pregnancies]...For my own conscience, knowing that my wife is not at risk and two, when she is injected the sperm will go straight into her womb than using other methods that could require us to try for longer period of time and then at the end of the day if your partner fails to take the pill or something happens she is put at risk. That is why l preferred semen washing. PREP is the one method I would never want for my negative partner to use and I do not see myself ever attempting to.”*  (021M, HIV-positive man, couple used ART/VL+SW) |
